# **RETRACTION:** A Long Non‐Coding RNA OLBC15 Promotes Triple‐Negative Breast Cancer Progression Via Enhancing ZNF326 Degradation

**DOI:** 10.1002/jcla.70169

**Published:** 2026-01-23

## Abstract

**RETRACTION**: DengC. , ZhangB. , ZhangY. , XuX. , XiongD. , ChenX. , and WuJ. , “A Long Non‐Coding RNA OLBC15 Promotes Triple‐Negative Breast Cancer Progression Via Enhancing ZNF326 Degradation,” Journal of Clinical Laboratory Analysis 34, no. 8 (2020): e23304, 10.1002/jcla.23304.32329931 PMC7439339

The above article, published online on 24 April 2020, in Wiley Online Library (http://onlinelibrary.wiley.com/), has been retracted by agreement between the authors; the journal Editor‐in‐Chief, Rong Fu; and Wiley Periodicals, LLC. The corresponding author Jiaojiao Wu contacted the journal to request that the article be retracted for the following reasons: 1. An inability to reproduce key experimental results; 2. Technical errors in the experimental protocols that may have compromised the reliability and reproducibility of the data (inconsistencies in RNA pulldown assay conditions, potential cross‐contamination in cell line experiments, and insufficient validation of antibody specificity for ZNF326 detection); and 3. Discrepancies in the original data, calling into question the western blot images in Figures 4B, 5A, and 5B; the quantification data for migration assays; and IHC scoring consistency in clinical samples. Following an investigation, images in Figures 4 and 5 were found to be duplicated from earlier publications: Figure 4D being from Figure 3A in Koo et al. 2017 (https://doi.org/10.1158/1535‐7163.MCT‐17‐0077), Figure 5B being from Figure 5B in Shen et al. 2019 (https://doi.org/10.1002/jcla.23122), and Figure 5E being from Figure 2G in Li et al. 2018 (https://doi.org/10.2147/CMAR.S183355). Due to the scope of these errors, the editor has lost confidence in the results reported, and therefore the article must be retracted.

**RETRACTION**: C. Deng , B. Zhang , Y. Zhang , X. Xu , D. Xiong , X. Chen , and J. Wu , “A Long Non‐Coding RNA OLBC15 Promotes Triple‐Negative Breast Cancer Progression Via Enhancing ZNF326 Degradation,” Journal of Clinical Laboratory Analysis 34, no. 8 (2020): e23304, 10.1002/jcla.23304.32329931 PMC7439339

The above article, published online on 24 April 2020, in Wiley Online Library (http://onlinelibrary.wiley.com/), has been retracted by agreement between the authors; the journal Editor‐in‐Chief, Rong Fu; and Wiley Periodicals, LLC. The corresponding author Jiaojiao Wu contacted the journal to request that the article be retracted for the following reasons: 1. An inability to reproduce key experimental results; 2. Technical errors in the experimental protocols that may have compromised the reliability and reproducibility of the data (inconsistencies in RNA pulldown assay conditions, potential cross‐contamination in cell line experiments, and insufficient validation of antibody specificity for ZNF326 detection); and 3. Discrepancies in the original data, calling into question the western blot images in Figures 4B, 5A, and 5B; the quantification data for migration assays; and IHC scoring consistency in clinical samples. Following an investigation, images in Figures 4 and 5 were found to be duplicated from earlier publications: Figure 4D being from Figure 3A in Koo et al. 2017 (https://doi.org/10.1158/1535‐7163.MCT‐17‐0077), Figure 5B being from Figure 5B in Shen et al. 2019 (https://doi.org/10.1002/jcla.23122), and Figure 5E being from Figure 2G in Li et al. 2018 (https://doi.org/10.2147/CMAR.S183355). Due to the scope of these errors, the editor has lost confidence in the results reported, and therefore the article must be retracted.

